# Adjuvant External Beam Radiotherapy Reduces Local Recurrence in Poorly Differentiated Thyroid Cancer: A Multicenter Retrospective Cohort Study Describing Outcomes in the Treatment of Resectable Poorly Differentiated Thyroid Cancer

**DOI:** 10.1245/s10434-025-17434-2

**Published:** 2025-05-23

**Authors:** Pascal K. C. Jonker, Jan H. Koetje, John Turchini, Jia Feng Alex Lin, Anthony J. Gill, Thomas Eade, Ahmad Aniss, Roderick Clifton-Bligh, Bettien M. van Hemel, Diana Learoyd, Hans H. G. Verbeek, Thera P. Links, Bruce Robinson, Venessa Tsang, Stanley Sidhu, Schelto Kruijff, Mark S. Sywak

**Affiliations:** 1 University of Sydney Endocrine Surgical Unit, Royal North Shore Hospital, Northern Sydney Local Health District, St. Leonards, Australia; 2 Department of Surgery, Division of Surgical Oncology, University Medical Center Groningen, University of Groningen, Groningen, The Netherlands; 3 Northern Clinical School, Sydney Medical School, Faculty of Medicine and Health, University of Sydney, Sydney, NSW Australia; 4 Douglass Hanly Moir Pathology, Macquarie Park, NSW Australia; 5 Discipline of Pathology, MQ Health, Macquarie University, Macquarie Park, NSW Australia; 6 Department of Anatomical Pathology, NSW Health Pathology, Royal North Shore Hospital, Northern Sydney Local Health District, St. Leonards, NSW Australia; 7 Cancer Diagnosis and Pathology Group, Kolling Institute of Medical Research, Royal North Shore Hospital, Northern Sydney Local Health District, St Leonards, NSW Australia; 8 Department of Radiation Oncology, Royal North Shore Hospital, Northern Sydney Local Health District, St Leonards, NSW Australia; 9 Department of Endocrinology, Royal North Shore Hospital, Northern Sydney Local Health District, St Leonards, Australia; 10 Department of Pathology, University Medical Center Groningen, University of Groningen, Groningen, The Netherlands; 11 Department of Radiotherapy, University Medical Center Groningen, University of Groningen, Groningen, The Netherlands; 12 Department of Internal Medicine, Division of Endocrinology, University Medical Center Groningen, University of Groningen, Groningen, The Netherlands

**Keywords:** Thyroid, Poorly differentiated, Cancer, PDTC, Turin, Radiotherapy, ^131^I-refractory, Surgery

## Abstract

**Background:**

Poorly differentiated thyroid carcinoma (PDTC) accounts for 5% of all thyroid cancers and is responsible for a large proportion of thyroid cancer-related deaths. The optimal treatment approach is not clear. This study aimed to evaluate the effect of postoperative intensity-modulated radiotherapy (IMRT) on the treatment of resectable PDTC. Additionally, treatment-related morbidity, characteristics of ^131^I-refractory disease, and factors affecting survival were assessed.

**Methods:**

The study included consecutive PDTC cases from 1997 to 2018, defined according to Turin criteria and treated in two tertiary referral centers. Surgery, IMRT, ^131^I, and systemic therapies were administered based on multidisciplinary team recommendations. The primary study outcome was 5-year local control after IMRT in cases with positive resection margins (micro- and macroscopic). The secondary outcomes were treatment-related morbidity within 30-days after completion of treatment (Clavien-Dindo and Common Terminology Criteria for Adverse Events [CTC-AE] 5.0), ^131^I-refractory disease characteristics using standardized definitions, and factors influencing survival.

**Results:**

Among 51 PDTC cases, 53% presented with metastatic disease. Adjuvant IMRT improved 5-year local control (100% vs. 17.5%; *p* = 0.02), with a higher number of grades 1 to 3 complications (*p* = 0.005) versus cases without IMRT. Within 13 months, ^131^I-refractory disease occurred in 62.7% of the patients and was more common in non-survivors (86.6% vs. 52.8%; *p* = 0.01). Positive resection margins and extrathyroidal extension were associated with poor survival in the univariate analysis, but were not significant in the multiple regression analysis.

**Conclusion:**

Adjuvant IMRT may reduce thyroid bed recurrence in resectable PDTC with positive resection margins, but is associated with increased treatment-related complications. ^131^I-refractory disease occurs frequently, with non-survivors progressing earlier to ^131^I resistance.

**Supplementary Information:**

The online version contains supplementary material available at 10.1245/s10434-025-17434-2.

Poorly differentiated thyroid cancer (PDTC) represents 0.3% to 8% of thyroid cancers.^1–3^ Between differentiated thyroid cancer (DTC) and anaplastic thyroid cancer (ATC), PDTC holds an intermediate status in 5-year overall survival (OS), disease-specific survival, and disease-free survival.^4–7^ The 5-year overall survival rate for PDTC, ranging from 62 to 85%, identifies it as the second most fatal thyroid cancer after ATC.^3,4,8–10^ The manner of death due to PDTC is locoregional disease recurrence and distant metastasis, responsible for 18% and 85% of deaths, respectively.^9,11^

The World Health Organization’s endocrine tumors classification adopts the Turin consensus, defining PDTC histopathologically based on growth patterns (solid, insular, or trabecular).^12^ Alternatively, Hiltzik’s classification, reliant on proliferative grading, is used in some high-volume centers.^13^ Hiltzik’s standard diagnoses more PDTC cases than Turin criteria in non-anaplastic follicular-cell derived thyroid cancers, but both have similar prognostic capacities.^14,15^

Standardized treatment guidelines for PDTC are lacking. The current strategy involves personalized, multidisciplinary management using surgery, radiotherapy, and systemic treatments tailored to individual patient and tumor characteristics. Decision-making is often based on DTC treatment guidelines, whereas the European Thyroid Association incorporates PDTC management in the advanced radioiodine (^131^I)-refractory thyroid cancer guidelines.^16–18^

Surgical resection with total thyroidectomy forms the cornerstone of treatment for resectable tumors, and the extent of nodal dissection depends on preoperative clinical or radiologic nodal involvement.^9^ The indications for postoperative radiotherapy remain controversial due to limited data, with no current available information on its impact on local and regional control.

When selected by age, T stage, and nodal status, postoperative radiotherapy in high-risk PDTC has been observed to have a positive impact on patient survival in some studies, whereas other studies have shown no survival benefit.^1,19,20^ The administration of ^131^I is recommended by the American Thyroid Association guidelines based on data suggesting that high-dosage (100–200 mCi) treatment is associated with improved survival.^1,16^ However, 50% of patients experience ^131^I-refractory PDTC when standardized criteria for the definition of ^131^I-refractory disease are applied.^21^ The true benefit of ^131^I in PDTC remains debatable due to inconsistencies in survival benefits, treatment variations, follow-up evaluations, and the various definitions used for ^131^I-refractory disease.^8–10,22,23^ Finally, comprehensive reporting of morbidity in all aspects of PDTC management is crucial for better-informed clinical decision-making. Outcome data capturing all treatment-related complications are scarce because of incomplete or focused reporting of complications related to individual treatment methods.^24–34^

This multicenter retrospective cohort study aimed to evaluate the effect of postoperative radiotherapy on local control of recurrent disease in the thyroid bed in resectable PDTC classified according to Turin criteria.^12^ In addition, we aimed to identify factors associated with disease-specific survival and provide insight into the incidence of ^131^I-refractory disease using a consensus definition. Finally, we aimed provide a comprehensive overview of morbidity related to surgery, radiotherapy, and systemic treatment.^35^

## Material and Methods

### Patient Population

This multicenter retrospective cohort study was conducted after approval from the local medical ethics committees at the University of Sydney (USYD) and the University Medical Center Groningen (UMCG). These Australian and Dutch hospitals are high-volume tertiary referral centers for endocrine pathology.

To identify patients with PDTC who underwent treatment at the USYD, a review of the databases in the Departments of Endocrine Surgery, Anatomical Pathology, and Radiation Oncology was performed using the diagnosis “poorly differentiated thyroid cancer” to identify patients treated between 1997 and 2018. Patients treated at the UMCG were identified by screening the histopathology reports of all patients undergoing thyroid surgery between 2006 and 2019. Patients with a histologically confirmed diagnosis of PDTC according to the Turin criteria with resectable thyroid tumors were included for analysis. All patients underwent histopathologic review of tumor tissue by an expert pathologist at the USYD or UMCG.

After diagnosis, patient-tailored treatments were discussed at multidisciplinary team meetings. The indication for ^131^I treatment was based on pretreatment imaging and histologic factors in all cases. When locoregional radiotherapy was incorporated into the treatment regimen, intensity-modulated radiotherapy (IMRT) with an intended dosage of 50 Gy or more was started. For some patients with progressive distant disease during the follow-up period, palliative systemic therapy with chemotherapy or targeted molecular therapy including tyrosine kinase inhibitors (TKIs) was initiated.

### Outcome Parameters

The primary aim of this study was to assess the beneficial effects of postoperative IMRT on 5-year local recurrence in the thyroid bed and disease-specific survival of patients with PDTC and positive resection margins. The secondary aims were to acquire insight into morbidity associated with surgery, IMRT, and systemic treatment; to characterize ^131^I-resistant disease; and to identify factors associated with poor survival.

### Definitions of the Inclusion Criteria and Outcome Variables

Patients with resectable, histopathologically confirmed PDTC were included for analysis. The diagnosis of PDTC was based on the Turin criteria.^8^ No high-grade differentiated thyroid carcinoma (HGDTC) cases were included.

The patients were restaged according to the eighth edition of the American Joint Committee on Cancer (AJCC)/tumor-node-metastasis (TNM) cancer-staging system. Restaging was performed using pre- and posttreatment ^131^I scans, neck-chest computed tomography (CT), whole-body positron emission tomography (PET)/CT scans with or without ultrasound, and magnetic resonance imaging (MRI) or bone scans performed within 3 months after the initial diagnosis. Positive resection margins were defined either macroscopically or microscopically. Tumor size was defined as the maximum primary tumor diameter reported on the final histology. The non-age-adjusted Charlson Comorbidity Index and Eastern Cooperative Oncology Group (ECOG) performance score were used to classify baseline comorbidity and performance status, respectively.

Treatment response was defined according to RECIST criteria and classified as local (thyroid bed), regional (locoregional lymph nodes neck levels 1 to 6), or distant response. Clavien-Dindo and Common Terminology Criteria for Adverse Events (CTC-AE) edition 5.0 were used to report all complications (including complications possibly related to disease progression) that occurred during or within 30 days after surgery, radiotherapy completion, or systemic treatment. Permanent recurrent laryngeal nerve (RLN) injury was defined as visualized non-functioning vocal cord 6 months after surgery, ongoing at the final follow-up evaluation, and confirmed on fiber-optic laryngoscopy. Temporary hypocalcemia was defined as a maximum of 6 months of calcium or vitamin D supplementation and persistent hypocalcemia as continuing supplementation after 6 months, ongoing at the final follow-up evaluation. The study defined ^131^I-refractory disease as no ^131^I uptake on a diagnostic ^131^I scan, no ^131^I uptake on a ^131^I scan performed several days after ^131^I therapy, ^131^I uptake present in some but not other tumor foci, PDTC metastasis progress despite ^131^I uptake, or PDTC metastasis progress despite cumulative ^131^I activity of more than 22.2 GBq (600 mCi).^35^

Disease-specific survival was defined as the time from initial diagnosis to final follow-up evaluation or death caused by PDTC. Persistent disease was defined as residual disease visualized by ultrasound or whole-body imaging (^131^I scans, CT, PET/CT scans, MRI, or bone scans).

### Local Intensity-Modulated Radiotherapy Protocols

All the patients treated at both centers underwent planning CT with the patient immobilized in a thermoplastic mask. At the USYD, an F-18 fluorodeoxyglucose (FDG)-PET scan was performed before radiation planning and fused to the planning CT. Gross tumor volume (GTV), high-risk clinical target volume (CTVHD), and low-risk volume (CTVLD) were defined. All the patients were planned to undergo multi-field IMRT or volumetric modulated arc therapy (VMAT) using a 5-mm setup margin and daily imaging (IGRT) for both fractions. Dose and fractionation ranged from 55 Gy in 20 fractions to 66 Gy in 30 fractions, most commonly 66 Gy (GTV), 60 Gy (CTVHD), and 54 Gy (CTVLD). All the patients were planned to have optimization of the dose off the larynx, trachea, and esophagus. When concurrent chemotherapy was used, it comprised docetaxel and carboplatin.

At the UMCG, preoperative imaging was fused with the planned CT, and GTV, CTVHD, and CTVLD were defined. Since 2015, patients have been treated primarily with VMAT using a 3-mm setup margin and daily IGRT for all fractions. The most common regimen was 66 Gy (CTVHD) and 52.8 Gy (CTVLD), delivered in 33 fractions with specific measures taken to optimize dosing on the parotid glands, submandibular glands, and swallowing structures.

### Statistical Analysis

Descriptive statistics were used to describe patient, tumor, and treatment characteristics. Differences between groups were assessed using Student’s *t* test, Mann-Witney *U* tests, and chi- square tests. Kaplan-Meier survival estimates with log-rank tests were performed to assess the effect of treatment on primary and secondary outcome measures. Multivariate analysis using Cox proportional-hazards model was performed to identify factors associated with disease-specific survival outcomes. The variables nodal status (c/pN1 vs. c/pN0), distant metastasis status (c/pM1 vs. c/pM0), tumor size (>40 vs. ≤40 mm), margin status (R2/R1 vs. R0), extrathyroidal extension (yes vs. no), vascular invasion (yes vs. no), treatment with chemoradiotherapy (yes vs. no), administration of TKIs (yes vs. no), and ^131^I-refractory status (yes vs. no) were included in the multivariate model. Significance was set at a *p* value lower than 0.05. The Statistical Package for the Social Sciences (SPSS, version 25; International Business Machines Corp, Armonk, NY, USA) and R were used for statistical analysis.

## Results

### Patient and Treatment Characteristics

The study enrolled 51 PDTC patients (38 USYD patients treated between 1997 and 2017; 13 UMCG patients treated between 2007 and 2018). A detailed description of the study population is provided in Table 1. The cohort had a median age of 67 years (range, 50–95 years), and 30 patients were female (59%). Of the 51 patients, 26 (51%) had a history of pre-existing multinodular goiter, and 3 (6%) reported previous exposure to radiation, including work in nuclear laboratories (*n* = 1), radiotherapy to the neck for lymphadenopathy of unknown origin (*n* = 1), and adjuvant radiotherapy as breast cancer treatment (*n* = 1).

**Table 1:** Demographics and histopathology characteristics of patients with poorly differentiated thyroid cancer

Parameter	Survivors (*n* = 36) *n* (%)	Non-survivors (*n* = 15) *n* (%)	Total (*n* = 51) *n* (%)	*p* Value
*General*				
Gender				0.76
Male	14 (38.9)	7 (46.7)	21 (41.2)	
Female	22 (61.1)	8 (53.3)	30 (58.8)	
Median age: years (range)	67 (20–95)	73 (55–84)	67 (50–95)	0.10
CCI				0.76
0	19 (52.8)	7 (46.7)	26 (51.0)	
≥1	17 (46.2)	8 (46.7)	25 (48.0)	
ECOG at diagnosis				0.05
0–1	34 (94.4)	11 (73,3)	45 (88,2)	
≥2	2 (5.6)	4 (26.7)	6 (11.8)	
Median follow-up: months (range)	45 (4–128)	31 (3–52)	36 (3–128)	0.02
*History and presentation*				
Thyroid history				0.57
Positive	20 (56.6)	8 (53.3)	28 (44.9)	
Negative	16 (44.4)	7 (46.7)	23 (45.1)	
Radiation exposure				0.73
Yes	3 (8.4)	0	3 (6.0)	
No	33 (91.7)	15 (100)	48 (94.0)	
Previous thyroid surgery				0.24
Yes	1 (2.8)	1 (6.7)	2 (4.0)	
No	35 (97.2)	14 (93.3)	49 (96.0)	
*Tumor characteristics*				
T stage				0.047
pT2	10 (36.1)	0	10 (19.6)	
pT3a	18 (50.0)	6 (40.0)	24 (47.1)	
pT3b	3 (8.3)	2 (13.3)	5 (9.8)	
pT4a	4 (11.1)	5 (33.4)	9 (17.6)	
pT4b	1 (2.8)	2 (13.3)	3 (5.9)	
N stage				0.99
c/pN0	24 (36.7)	10 (66.6)	34 (66.7)	
pN1a	3 (8.3)	1 (6.7)	4 (7.8)	
pN1b	9 (25.0)	4 (26.7)	13 (25.5)	
M stage				0.77
M0	18 (50.0)	6 (40.0)	24 (47.1)	
M1	18 (50.0)	9 (60.0)	27 (52.9)	
Median tumor size: mm (range)	50 (22–130)	60 (40–105)	50 (22–130)	0.78
Margin status				0.22
Negative	21 (58.3)	5 (33.3)	26 (51.0)	
Positive	15 (41.7)	10 (67.7)	25 (49.0)	
Extrathyroidal extension				0.003
Extensive	4 (11.1)	8 (53.3)	12 (23.5)	
Minimal	11 (30.5)	4 (26.7)	15 (29.4)	
Absent	21 (58.3)	3 (20.0)	24 (47.1)	
Capsular invasion				0.008
Unencapsulated	5 (13.9)	0	5 (9.8)	
Extensive	15 (41.7)	14 (93.3)	29 (56.9)	
Focal	10 (27.8)	1 (6.7)	11 (21.6)	
Absent^a^	6 (16.6)	0	6 (11.7)	
Vascular invasion				0.77
Extensive (≥4 foci)	4 (11.1)	1 (6.7)	5 (9.8)	
Focal (<4 foci)	4 (11.1)	1 (6.7)	5 (9.8)	
Absent	28 (77.8)	13 (86.6)	41 (80.4)	

CCI, non-age-adjusted Charlson Comorbidity Index; ECOG, Eastern Cooperative Oncology Group performance score

^**a**^All tumors without capsular invasion had vascular invasion.

The five most frequently reported symptoms at presentation were slow (>1 month; *n* = 19) or rapid (<1 month; *n* = 8) progression of a neck mass, neck pain (*n* = 8), voice changes (*n* = 5), and dysphagia (*n* = 5). In eight patients (16%), PDTC presented as an asymptomatic incidental lesion, whereas five patients (10%) presented with symptoms of metastatic disease. The main metastatic sites at diagnosis were lungs (*n* = 20), bone (*n* = 17), and hilar lymph nodes (*n* = 4).

After diagnosis, the patients received treatment in accordance with the recommendations of the multidisciplinary team, following international guidelines available at the time (Table 2). The median follow-up period was 36 months (range, 3–128 months). One patient progressed from PDTC to ATC within 3 months after the diagnostic hemithyroidectomy and was treated with subsequent debulking surgery. Most of the patients underwent a nodal dissection in addition to thyroidectomy, either prophylactic (*n* = 16) or therapeutic (*n* = 14). One patient received neo-adjuvant radiotherapy for unresectable PDTC, which resulted in a good response and subsequently enabled total thyroidectomy. A median dosage of 62 Gy (range, 30–70 Gy) adjuvant IMRT was administered to 8 of 24 patients with (micro- or macroscopic) positive resection margins. All IMRT treatments were completed as planned. Of the 51 patients, 3 received no adjuvant ^131^I. The reasons for abstaining from ^131^I were no uptake on the pre-ablation ^131^I scan, successful locoregional control with surgery and radiotherapy for a patient without metastatic disease, and disease progression with ATC in one patient after diagnostic hemithyroidectomy.

**Table 2:** Treatment characteristics

Parameter	Survivors (*n* = 36) *n* (%)	Non-survivors (*n* = 15) *n* (%)	Total (*n* = 51) *n* (%)	*p* Value
^*131*^*I-refractory*				
No	16 (44.4)	1 (6.7)	17 (33.3)	0.01
Yes	19 (52.8)	13 (86.6)	32 (62.7)	
Unknown	1 (2.8)	1 (6.7)	2 (4.0)	
Median ^131^I-refractory time from diagnosis: months (range)	25.5 (2.0–125.0)	9.0 (1.0–52.0)	12.5 (5.3–24.0)	0.003
Median ^131^I treatments to refractory: *n* (range)	2 (0–4)	1.0 (1–3)	1 (0–4)	0.32
Median ^131^I treatments from refractory: *n* (range)	1 (0–3)	0 (0–4)	0 (0–4)	0.03
Median ^131^I dosage to refractory: MBq (range))	11.1 (0–22.0)	5.6 (2.0–16.0)	7.8 (5.6–14.5)	0.09
Median ^131^I dosage from refractory: MBq (range)	6.1 (0–18)	0 (0–21)	0 (0–21.0)	0.02
*Treatment characteristics*				
Index thyroid surgery				0.57
Hemithyroidectomy	0	2 (13.3)	2 (4.0)	
Total thyroidectomy	36 (100)	13 (86.7)	49 (96.0)	
Neck dissection				0.54
Perithyroidal lymph nodes	2 (5.6)	1 (6.7)	3 (5.9)	
Central compartment dissection	10 (27.8)	3 (20)	13 (25.5)	
Central and unilateral neck dissection	5 (13.8)	5 (33.3)	10 (19.6)	
Central and bilateral neck dissection	3 (8.4)	1 (6.7)	4 (7.8)	
No lymph node dissection	16 (44.4)	5 (33.3)	21 (41.2)	
(Neo)-adjuvant IMRT				0.72
Yes	6 (16.7)	3 (20.0)	9 (17.6)	
No	30 (83.3)	12 (80.0)	42 (82.4)	
Median adjuvant IMRT dosage on thyroid bed: Gy (range)	60 (30–66)	66 (55–70)	62 (30–70)	0.54
Adjuvant ^131^I				0.78
Yes	34 (94.4)	14 (93.3)	48 (94.2)	
No	2 (5.6)	1 (6.7)	3 (5.8)	
Median no. of treatments (range)	2 (0–6)	1 (0–7)	2 (0–7)	
Median dosage: MBq (range)	11 (0–36)	6 (0–37)	10 (0–37)	0.27
Adjuvant chemotherapy				0.17
Yes	3 (8.3)	4 (26.7)	7 (13.7)	
No	33 (91.7)	11 (73.3)	44 (86.3)	
Adjuvant TKI				0.42
Yes	5 (13.9)	4 (26.7)	9 (17.6)	
No	31 (86.1)	11 (73.3)	42 (82.4)	

^131^I, radioiodine; MBq, mega becquerel; IMRT, intensity-modulated radiotherapy; TKI, tyrosine kinase inhibitor

For 13 patients, systemic treatment with chemotherapy (*n* = 6) or tyrosine kinase inhibitors (*n* = 7) was started to treat distant progression (*n* = 10), to be a radiosensitizer (*n* = 2), or to treat a secondary malignancy (*n* = 1). The patients undergoing treatment with targeted molecular therapy received lenvatinib (*n* = 5) or sorafenib (*n* = 2). For five patients, a switch of systemic treatment was made to another type of chemotherapy (*n* = 2) or lenvatinib (*n* = 3) due to progressive disease. None of the systemic treatment regimens was stopped due to side effects.

### Primary Outcome Measures

**Fig. 1:**
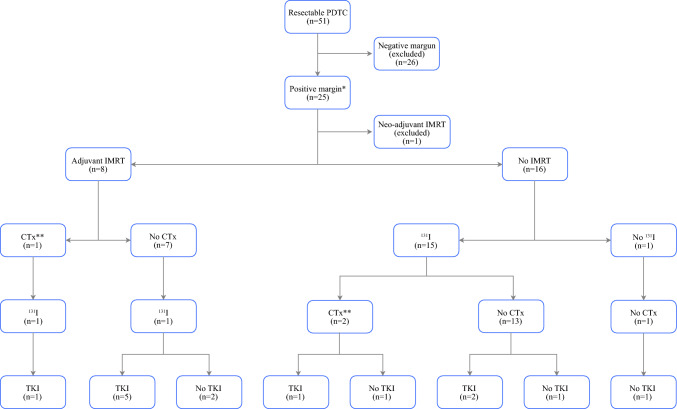
*, microscopic or macroscopic involved margins; **, Chemosensitizer during radiotherapy; ***,1 patient received RCVP for non-hodgkin lymphoma and no chemotherapy for PDTC. Following RCVP, lenvatinib was initiated for progressive 131I refractory PDTC. Abbreviations: PDTC, poorly differentiated thyroid cancer; IMRT, intensity modulated radiotherapy; CTx, chemotherapy; 131I, adjuvant radioiodine; TKI, tyrosine kinase inhibitor

**Fig. 2:**
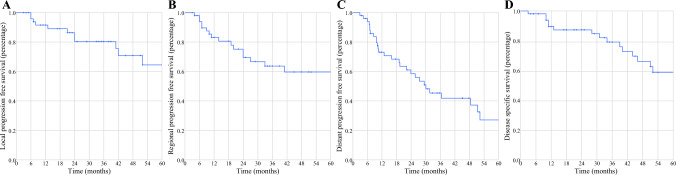
Overview of local (**A**), regional (**B**) and distant (**C**) progression free survival of 51 patients with poorly differentiated thyroid cancer included for the analysis. The disease specific survival of the cohort is depicted in Fig. 2**D**

*Local Control and Disease-Specific Survival of Patients With Positive Resection Margins.* The flowchart in Fig. 1 provides an overview of the treatment regimens for 24 patients with positive resection margins (micro- or macroscopic) treated with either adjuvant IMRT or no radiotherapy. The patients at the USYD who underwent IMRT were treated between 2009 and 2018, whereas those at the UMCG received IMRT between 2015 and 2019 (Fig. 2).

**Fig. 3:**
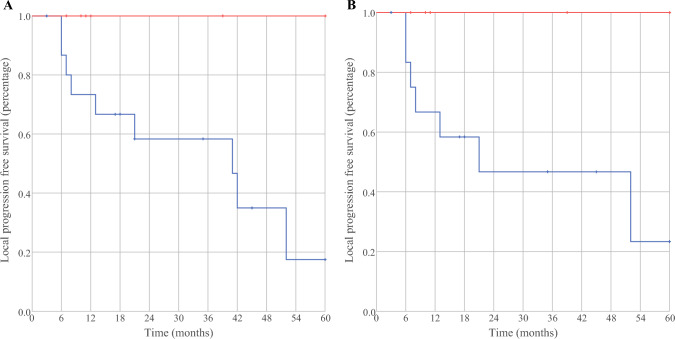
**A** shows an improvement (*p* = 0.02) in 5-year local progression free survival of 8 patients with a positive resection margin that underwent postoperative intensity modulated radiotherapy (IMRT, red line) versus patients with a positive resection margin without adjuvant IMRT (*n* = 16, blue line). **B** confirms the beneficial effect of IMRT followed by adjuvant treatment with TKI’s (*p* = 0.04) in a subcategory of patients with a positive resection margin treated with IMRT on the thyroid bed and adjuvant TKI’s for progressive distant metastasis (*n* = 6) versus patients not treated with IMRT and TKI’s (*n* = 13)

For the patients with positive resection margins, the follow-up evaluation was similar between those treated with IMRT (26 months; range, 10–88 months) and those treated without IMRT (33 months; range, 3–128 months) (*p* = 0.86). Adjuvant IMRT improved the 5-year local control rate (100% vs. 17.5%; *p* = 0.02; Fig. 3a), but did not affect 5-year disease-specific survival (56.3% vs. 44.4%; *p* = 0.83) for the patients with positive resection margins compared with the patients not undergoing adjuvant IMRT. The rates of positive (micro- or macroscopic) resection margins were equal, and neither the demographics nor the tumor characteristics differed between the two groups (Table 3). The patients treated with adjuvant IMRT received more TKIs than the patients treated without adjuvant IMRT (*p* = 0.002; Table 3). A subgroup analysis confirmed the improved 5-year local control rate for the patients with positive resection margins undergoing adjuvant IMRT and receiving TKI compared with the patients without adjuvant IMRT and with no TKI treatment (*p* = 0.04; Fig. 3b).

**Table 3:** Characteristics of patients with positive resection margins treated with and without intensity-modulated radiotherapy

Parameter	IMRT (*n* = 8) *n* (%)	No IMRT (*n* = 16) *n* (%)	Total (*n* = 24) *n* (%)	*p* Value
*General*				
Gender				0.14
Male	5 (62.5)	5 (31.3)	10 (41.7)	
Female	3 (37.5)	11 (68.8)	14 (58.3)	
Median age: years (range)	60.5 (44–67)	72.5 (21–83)	67.0 (21–83)	0.05
CCI				0.41
0	4 (50.0)	5 (31.3)	9 (37.5)	
≥1	4 (50.0)	11 (68.8)	15 (58.3)	
ECOG at diagnosis				0.13
0–1	8 (100)	11 (68.8)	19 (79.2)	
≥2	0	5 (31.3)	5 (20.7)	
Median follow-up: months (range)	26 (10–88)	33 (3–128)	33 (3–128)	0.86
*Tumor characteristics*				
T stage				0.79
pT2	0	3 (18.8)	3 (12.5)	
pT3a	2 (25.0)	4 (25.0)	6 (25.0)	
pT3b	3 (37.5)	1 (6.3)	4 (16.7)	
pT4a	3 (37.5)	5 (31.3)	8 (33.3)	
pT4b	0	3 (18.8)	3 (12.5)	
N stage				0.26
c/pN0	2 (25.0)	10 (62.5)	12 (50.0)	
pN1a	1 (12.5)	2 (12.5)	3 (12.5)	
pN1b	5 (62.5)	4 (25.0)	9 (37.5)	
M stage				
M0	2 (25.0)	8 (50.0)	10 (41.7)	0.34
M1	6 (75.0)	8 (50.0)	14 (68.3)	
Median tumor size: mm (range)	68 (40–130)	60 (22–125)	60 (22–130)	0.40
Margin status				0.48
R1	7 (87.5)	12 (75.0)	19 (79.2)	
R2	1 (12.5)	4 (25.0)	5 (20.8)	
Extrathyroidal extension				0.78
Extensive	3 (37.5)	7 (43.8)	12 (50.0)	
Minimal	4 (50.0)	9 (56.2)	11 (45.8)	
Absent	1 (12.5)		1 (4.2)	
Capsular invasion				0.99
Unencapsulated	1 (12.5)	2 (12.5)	3 (12.5)	
Extensive	6 (75.0)	12 (75.0)	18 (75.0)	
Focal	1 (12.5)	2 (12.5)	3 (12.5)	
Vascular invasion				0.72
Extensive (≥4 foci)	7 (87.5)	14 (87.5)	21 (87.5)	
Focal (<4 foci)	1 (12.5)	2 (12.5)	3 (12.5)	
*Treatment characteristics*				
Index thyroid surgery				0.69
Hemithyroidectomy	0	1 (6.3)	1 (4.2)	
Total thyroidectomy	8 (100)	15 (93.7)	13 (95.8)	
Neck dissection				0.32
Yes	7 (87.5)	11 (68.7)	18 (75.0)	
No	1 (12.5)	5 (31.3)	6 (25.0)	
Adjuvant radiotherapy				N/A
Yes	8 (100)	0	8 (33.3)	
No	0	16 (100)	16 (66.7)	
Adjuvant ^131^I				0.47
Yes	8 (100)	15 (93.7)	23 (95.8)	
No	0	1 (6.3)	1 (4.2)	
Adjuvant chemotherapy				0.57
Yes	1 (12.5)	2 (12.5)	4 (16.7)	
No	7 (87.5)	14 (87.5	20 (83.3)	
Adjuvant TKI				
Yes	6 (75.0)	2 (12.5)	8 (32.0)	0.002
No	2 (25.0)	14 (87.5)	16 (68.0)	
*Median complications (range)*				
Grade 1	2 (1–9)	0 (0–6)	1 (0–9)	0.005
Grade 2	4 (2–6)	2 (0–6)	2 (0–6)	0.02
Grade 3	1 (0–5)	0 (0–5)	1 (0–5)	0.04
Grade 4	0 (0–0)	0 (0–1)	0 (0–1)	0.48
Grade 5	0 (0–0)	0 (0–1)	0 (0–1)	0.31
Total	8 (5–14)	2 (0–14)	4 (0–14)	0.01

IMRT, intensity modulated radiotherapy; CCI, non-age-adjusted Charlson Comorbidity Index; ECOG, Eastern Cooperative Oncology Group performance score; TKI, tyrosine kinase inhibitor

### Secondary Outcome Measures

*Treatment-Related Morbidity.* Initial surgery was uneventful in 29 cases (56.9%). In the remaining 22 cases, 32 complications were registered (Table S1). Grade 2 complications occurred most often (65.6%), whereas no grade 4 or 5 complications were observed. In 4 of the 51 cases, RLN dysfunction was documented, consisting of three permanent injuries (5.9%) and one temporary injury (2.0%). In addition, the RLN was resected in two cases due to macroscopic tumor involvement (3.9%). Hypocalcemia consisted of 2 permanent (3.9%) and 12 temporary (23.5%) cases.

The patients with positive resection margins who underwent adjuvant IMRT had significantly more complications than the patients without adjuvant IMRT (*p* = 0.005). All the patients undergoing IMRT reported complications associated with radiotherapy. This was attributable to grade 1 (*p* = 0.005), grade 2 (*p* = 0.02), and grade 3 (*p* = 0.04) complications. No grade 4 or 5 complications were registered. Two grade 5 complications were registered in patients who had positive resection margins without adjuvant IMRT treatment.

One patient died of respiratory insufficiency due to pseudomonas pneumonia during palliative radiotherapy for spinal metastasis. A second patient with pre-existing dysfunction of the left RLN consented not to have a tracheostoma preoperatively and died after a right recurrent nerve injury during resection of a paratracheal mass with wedge resection of the right lung for recurrent PDTC.

The initially administered systemic treatment was uneventful for 3 (23%) of 13 patients, whereas 26 complications were registered in the remaining 10 patients. Grade 2 complications attributed to 50% of the adverse events (*n* = 13) and no grade 4 or 5 events related to systemic treatment occurred. An overview of all complications per treatment entity, including palliative treatment, for the cohort is provided in Tables S1, S2 and S3.

### *Characteristics of *^*131*^*I-Resistant Disease*

The majority of the patients (62.7%) included for analysis in this study had ^131^I-refractory disease diagnosed at presentation or during follow-up evaluation (Table 2). The median time from initial diagnosis to the development of ^131^I-refractory disease was 12.5 months (range, 5.3–24.0 months). The patients received a median of one ^131^I treatment (range, 0–4 treatments) before the development of ^131^I resistance. The rate of ^131^I-resistant PDTC was higher for the patients who died of their disease than for those who remained alive (86.6% vs. 52.8%; *p* = 0.01). The non-survivors had progressed faster toward ^131^I resistance (9.0 vs. 25.5 months; *p* = 0.003). The survivors received more ^131^I treatments after diagnosis of ^131^I-resistant disease than the non-survivors (1 vs. 0 treatments; *p* = 0.03).

### Survival

The 5-year local, regional, and distant progression-free survival rates of the cohort were respectively 64.5%, 59.7% and 27.2% (Fig. 2a–c). The 5-year disease-specific survival was 58.8% (Fig. 2d). At the final follow-up evaluation, 17 patients (33.3%) were alive without evidence of disease, 20 patients (39.2%) had died, and 14 patients (27.5%) were alive with persistent disease. In the univariate regression analysis, poor disease-specific survival in PDTC was associated with positive margin status (hazard ratio [HR], 6.06; 95% confidence interval [CI], 1.32–27.81; *p* = 0.03) and extrathyroidal extension (HR, 11.43; 95% CI 1.47–88.74; *p* = 0.02). No significant association with disease-specific survival was found in the multiple regression analysis (Table 4).

**Table 4:** Uni- and multivariate analysis

	Disease-specific survival			
	Univariate		Multivariate	
Variable	HR (95% CI)	*p* Value	HR (95% CI)	*p* Value
Nodal status (c/pN1 vs. c/pN0)	1.06 (0.31–3.61)	0.92	N/A	N/A
Distant status ( c/pM1 vs. c/pM0)	0.63 (0.20–2.01)	0.43	N/A	N/A
Tumor size (>40 vs. ≤40 mm)	1.46 (0.19–11.49)	0.72	N/A	N/A
Margin status (R2/R1 vs. R0)	6.06 (1.32–27.81)	0.03	0.94 (0.12–7.54)	0.95
Extrathyroidal extension (yes vs. no)	11.43 (1.47–88.74)	0.02	12.14 (0.75–195.60)	0.08
Vascular invasion (yes vs. no)	1.46 (0.18–11.49)	0.34	N/A	N/A
IMRT/CTx (yes vs. no)	0.33 (0.04–2.66)	0.32	N/A	N/A
TKI (yes vs. no)	1.06 (0.37–3.02)	0.91	N/A	N/A
^131^I-refractory (yes vs. no)	5.32 (0.68–41.6)	0.11	N/A	N/A

HR, hazard ratio; CI, confidence interval; N/A, not applicable; IMRT, intensity-modulated radiotherapy; CTx, systemic treatment; TKI, tyrosine kinase inhibition; ^131^I, radioiodine

## Discussion

This multicenter retrospective cohort study addressed several aspects in the management of patients with resectable PDTC diagnosed according to the Turin criteria. We observed improved local control in the thyroid bed of patients with PDTC and positive resection margins treated with adjuvant IMRT. The univariate analysis identified positive margin status and extrathyroidal extension as factors associated with poor disease-specific survival in PDTC. However, these factors were not significant in the multiple regression analysis, suggesting that other variables also may contribute to disease-specific survival in PDTC. In 62.7% of the patients, ^131^I resistance was found either at diagnosis or during follow-up evaluation. The patients who died of their disease had earlier development of ^131^I resistance than those who remained alive at the end of the study. Whereas surgery and systemic treatment have relative limited morbidity, adjuvant IMRT is associated with an increased complication rate, particularly grades 1 to 3 complications.

This study demonstrated that patients with resectable PDTC exhibit a varied initial presentation, spanning from asymptomatic incidental findings to symptomatic metastatic disease. Notably, metastatic disease was present in 53% of the patients at the time of diagnosis, a substantially higher rate than in other case studies reporting rates between 26 and 33%.^9,21^ Our 5-year disease-specific survival of 59% was lower than previously documented, typically between 62 and 85%.^3,4,8–10^ The lower survival rate is especially remarkable because other studies also included cases with non-resectable PDTC. This reduced survival might be attributable to the relative high proportion of patients presenting with metastatic disease.

Another finding from this study was the reduction of local recurrence among patients with positive resection margins who received postoperative IMRT. Whereas an overall survival benefit of IMRT for selected high-risk patients with PDTC was suggested previously, our data could not confirm this for tumors with positive resection margins.^19^ We found that for 63% of patients, ^131^I-resistant PDTC developed within 13 months after the initial diagnosis, a considerably higher rate than the previously reported 50% using a standard definition for ^131^I-refractory disease.^21^ Our findings further confirmed the high-risk characteristics of PDTC compared with more indolent histologic subtypes of thyroid cancer.

The retrospective nature of our study introduced inherent biases, including selection bias and incomplete data capture such as no complication data of ^131^I treatment. The use of medical records and historical data could have led to variations in quality and completeness of information across cases. In addition, the absence of PDTC treatment guidelines and the low incidence of the disease posed a challenge because treatment of this small cohort was based on individualized approaches discussed in multidisciplinary teams of high-volume tertiary referral centers. This may have contributed to variability in outcomes and may have complicated the identification of prognostic factors. The study focused on early morbidity. No information on long-term toxicities of IMRT and chemotherapy was provided. Finally, ^131^I-related morbidity was not included in this study due to insufficient reporting in clinical files. In addition, because uniform tumor genotyping (e.g., RAS, BRAF, TP53, TERT) was not systematically performed for our retrospective cohort, future multicenter prospective registries should address this gap to clarify genotype–phenotype relationships in PDTC. Recognizing these limitations is crucial for a nuanced interpretation of this study’s findings.

We found a reduced local recurrence rate for the patients with positive resection margins who underwent adjuvant IMRT. However, most of the patients also received adjuvant lenvatinib for progressive metastatic disease. The reduced recurrence rate in the thyroid bed may have been partially linked to the administration of adjuvant lenvatinib. The potential beneficial effect of lenvatinib in PDTC is illustrated by subgroup analysis in the SELECT trial showing a progression-free survival of 14.8 months during treatment with lenvatinib compared with 2.1 months in the placebo group.^24^ This is further supported by subsequent data confirming an improved progression-free survival after administration of lenvatinib with or without pembroluzimab.^25–34^ These novel agents are likely to become part of PDTC management in the near future. Preclinical findings suggest that lenvatinib may have a synergistic effect on radiotherapy by reducing thyroid cancer growth in papillary thyroid cancer (PTC) cell lines.^36^ However no (pre)clinical studies have specifically addressed a synergistic effect of IMRT and TKIs in PDTC. In our cohort, TKIs were administered in an adjuvant setting primarily for progressive disease, emphasizing the need for prospective, multicenter trials to investigate this potential synergy despite the challenges posed by PDTC’s low incidence. This paves the way for discerning the individual effects of IMRT and novel forms of systemic treatment on local recurrence PDTC. Future research should assess the clinical benefits and toxicities of proton therapy as well as long-term treatment-related toxicities and quality of life in relation to the chosen treatment.

Based on the results of our study, adjuvant IMRT might be considered to reduce thyroid bed recurrence rates for patients with resectable PDTC and positive resection margins. The absence of significant differences in margin status, age, or ECOG between the IMRT group and the non-IMRT group underscores that real-world decision-making for IMRT in PDTC is highly nuanced, reflecting both multidisciplinary input and consideration of patient- and disease-specific factors. During the process of shared decision-making, the potential benefit of adjuvant IMRT should be carefully balanced against patient factors and the associated early treatment morbidity.

Our study confirmed the poor disease-specific survival of patients with PDTC, but the underlying mechanisms remain unclear. Distant metastasis, pT4a disease, and extrathyroidal extension were previously linked to poor survival.^9,21^ In addition, ^131^I resistance may be a driver of progressive distant metastases and subsequent poor survival in PDTC. The potential significance of ^131^I resistance as a factor affecting survival is further supported by the poor response rates after ^131^I treatment, expressed by persistent disease and no survival benefit.^9^ Patients with early progression toward ^131^I resistance are likely to have a more aggressive form of PDTC. These patients may benefit from targeted systemic treatment regiments or immunotherapy to improve progression-free survival versus treatment with ^131^I only.^24–34^ Additional data using standardized definitions of ^131^I-refractory PDTC are required for accurate assessment of its significance and the subsequent implications for the inclusion of ^131^I and systemic treatment in the PDTC treatment protocol.

In conclusion, adjuvant IMRT improves local control for patients with PDTC and positive resection margins but increases treatment-related morbidity. In the majority of patients, ^131^I resistance develops, with survivors showing delayed progression toward ^131^I resistance. Surgery and systemic treatment come with relatively low morbidity. The low incidence of this rare disease shows the urgent need for an international consortium similar to the Turin group focusing on the establishment of a knowledge agenda and collaborative prospective clinical studies.

## Supplementary Information

Below is the link to the electronic supplementary material.

Supplementary file1 (DOCX 42 KB)
