# Supplementary material for: Adjuvant External Beam Radiotherapy Reduces Local Recurrence in Poorly Differentiated Thyroid Cancer: A Multicenter Retrospective Cohort Study Describing Outcomes in the Treatment of Resectable Poorly Differentiated Thyroid Cancer
Source: Ann Surg Oncol. 2025 May 23;32(9):6752–64. doi: 10.1245/s10434-025-17434-2 (PMC12317901; doi:10.1245/s10434-025-17434-2)
Supplement: Supplementary file 1 — Supplementary file1 (DOCX 42 KB) [file 10434_2025_17434_MOESM1_ESM.docx]

**Supplementary material**

**Supplementary table 1 –** Surgical complication overview

| **Complication**  **grade** | **R0 without IMRT**  **(26 patients)** | | **R1/R2 and IMRT**  **(8 patients)** | | **R1/R2 without IMRT**  **(16 patients)** | | **R1/R2 and neo-adjuvant IMRT**  **(1 patient)** | |
| --- | --- | --- | --- | --- | --- | --- | --- | --- |
| **Grade 1** | **Complications (n)** | **Description** | **Complications**  **(n)** | **Description** | **Complications**  **(n)** | **Description** | **Complications**  **(n)** | **Description** |
| *Initial surgery* | 1 | RLN damage left, permanent | 3 | Dysphagia  Hoarseness  Seroma | 1 | RLN damage, temporary | 0 | N/A |
| *Palliative surgery* | 0 | N/A | 0 | N/A | 0 | N/A | 0 | N/A |
| **Grade 2** |  |  |  |  |  |  |  |  |
| *Initial surgery* | 6 | Dyspnea  Hypocalcemia, permanent  Hypocalcemia, temporary (4) | 5 | Hypocalcemia, permanent  Hypocalcemia, temporary (3)  Nausea | 10 | Anemia (2)  Dyspnea (2)  Hypocalcemia, temporary (5)  RLN damage right, permanent (resection) | 0 | N/A |
| *Palliative surgery* | 1 | Urinary tracrt infection | 1 | Hypocalcemia, temporary | 2 | Atrial flutter  Pain, shoulder | 0 | Not applicable |
| **Grade 3** |  |  |  |  |  |  |  |  |
| *Initial surgery* | 1 | Permanent right RLN damage | 3 | Tracheal perforation  RLN resection left  RLN damage right, permanent | 2 | Atrial fibrillation  Urine retention | 0 | N/A |
| *Palliative surgery* | 4 | Bleeding (2)  Wound infection  Osteomyelitis | 2 | Urinary obstruction  Pyelonefritis | 6 | Carotid damage, iatrogenic  Esophageal fistula  Bleeding, intraoperative  Septic artritis  Urinary tract obstruction  Pyelonefritis | 0 | N/A |
| **Grade 4** |  |  |  |  |  |  |  |  |
| *Initial surgery* | 0 | N/A | 0 | N/A | 0 | N/A | 0 | N/A |
| *Palliative surgery* | 0 | N/A | 0 | N/A | 0 | N/A | 0 | N/A |
| **Grade 5** | 0 | N/A |  |  |  |  |  |  |
| *Initial surgery* | 0 | N/A | 0 | N/A | 0 | N/A | 0 | N/A |
| *Palliative surgery* | 0 | N/A | 0 | N/A | 1 | RLN damage right, permanent | 0 | N/A |

Supplementary table 1 – *legend*

*IMRT = intensity modulated radiotherapy; N/A = not applicable; RLN = recurrent laryngeal nerve; temporary hypocalcemia = maximum of 6 months suppletion; temporary hypocalcemia = continuing suppletion after 6 months, ongoing at final follow-up; R0 = microscopically negative resection margin; R1 = microscopically involved margin; R2 = macroscopic involved margins*

**Supplementary table 2** – Radiotherapy complication overview

| **Complication grade** | **R0 without IMRT**  **(26 patients)** | | **R1/R2 and IMRT**  **(8 patients)** | | **R1/R2 without IMRT**  **(16 patients)** | | **R1/R2 and neo-adjuvant IMRT**  **(1 patient)** | |
| --- | --- | --- | --- | --- | --- | --- | --- | --- |
| **Grade 1** | **Complications (n)** | **Description** | **Complications**  **(n)** | **Description** | **Complications**  **(n)** | **Description** | **Complications**  **(n)** | **Description** |
| *Index IMRT* | 4 | Dysphagia  Fatigue (2)  Odynophagia | 15 | Dermatitis (2)  Dysphonia  Dysphagia  Fatigue  Headache  Hoarseness (2)  Lymphedema  Mucositis  Neck edema (2)  Vomiting  Weight loss  Xerostomia | 8 | Cough  Dysphagia (2)  Dysphonia  Fatigue (2)  Mucus | 2 | Mucositis  Dermatitis |
| *Palliative radiotherapy* | 0 | N/A | 0 | N/A | 3 | Dermatitis  Fatigue  Ulcera, pressure | N/A | N/A |
| **Grade 2** |  |  |  |  |  |  |  |  |
| *Index IMRT* | 1 | Mucositis | 13 | Cough  Dermatitis (5)  Dysphagia (2)  Itching  Oesophagitis (2)  Pain  Rash | 11 | Constipation  Dermatitis (2)  Dysphagia (3)  Mucositis (2)  Pain  Weight loss  Xerostomia | 2 | Dysphagia  Fatigue |
| *Palliative radiotherapy* | 0 | N/A | 1 | Upper respiratory infection | 6 | Candida  Delirium  Pain, troath  Pain, metastasis  SIADH  Thrombosis | N/A | N/A |
| **Grade 3** |  |  |  |  |  |  |  |  |
| *Index IMRT* | 9 | Anorexia (2)  Dermatitis  Fatigue (2)  Nausea (2)  Constipation (2) | 3 | Esophageal stricture  RLN damage right, permanent  Sepsis | 1 | Stridor | 0 | N/A |
| *Palliative radiotherapy* | 0 | N/A | 1 | Obstipation | 2 | Pneumonia  Pancytopenia | N/A | N/A |
|  |  |  |  |  |  |  |  |  |
| **Grade 4** |  |  |  |  |  |  |  |  |
| *Index IMRT* | 0 | N/A | 0 | N/A | 0 | N/A | 0 | N/A |
| *Palliative radiotherapy* | 0 | N/A | 0 | N/A | 1 | Respiratory insufficiency | N/A | N/A |
| **Grade 5** | 0 | N/A |  |  |  |  |  |  |
| *Index IMRT* | 0 | N/A | 0 | N/A | 0 | N/A | 0 | N/A |
| *Palliative radiotherapy* | 0 | N/A | 0 | N/A | 1 | Death (respiratory insufficiency) | N/A | N/A |

Supplementary table 2 *– legend*

*IMRT = intensity modulated radiotherapy; N/A = not applicable; R0 = microscopically negative resection margin; R1 = microscopically involved margin; R2 = macroscopic involved margins*

**Supplementary table 3 –** Systemic treatment complication overview

| **Complication grade** | **R0 without IMRT**  **(26 patients)** | | **R1/R2 and IMRT**  **(8 patients)** | | **R1/R2 without IMRT**  **(16 patients)** | | **R1/R2 and neo-adjuvant IMRT**  **(1 patient)** | |
| --- | --- | --- | --- | --- | --- | --- | --- | --- |
| **Grade 1** | **Complications (n)** | **Description** | **Complications**  **(n)** | **Description** | **Complications**  **(n)** | **Description** | **Complications**  **(n)** | **Description** |
| *First systemic therapy* | 2 | Alopecia  Fatigue | 7 | Abdominal pain (2)  Fatigue (2)  Nausea  Oral ulceration  Proteinuria | N/A | N/A | N/A | N/A |
| *Additional systemic therapy* | 0 | N/A | 0 | N/A | 1 | Neutropenia | N/A | N/A |
| **Grade 2** |  |  |  |  |  |  |  |  |
| *First systemic therapy* | 3 | Cushing  Reflux  Trombocytopenia | 10 | Artralgia  Diarrhea  Hand-feet syndrome  Hypertension (2)  Hypocalcemia  Pain, troath  Rectal bleeding  Stomatitis  Urinary tract infection | N/A | N/A | N/A | N/A |
| *Additional systemic therapy* | 0 | N/A | 1 | Nausea | 2 | Alopecia  Dysphagia | N/A | N/A |
| **Grade 3** |  |  |  |  |  |  |  |  |
| *First systemic therapy* | 1 | Convulsion | 3 | Blistering, feet  Vomiging  Tongue pain | N/A | N/A | N/A | N/A |
| *Additional systemic therapy* | 0 | N/A | 1 | Intracerebral bleeding metastasis (lenvatinib) | 1 | Dermatitis | N/A | N/A |
| **Grade 4** |  |  |  |  |  |  |  |  |
| *First systemic therapy* | 0 | N/A | 0 | N/A | N/A | N/A | N/A | N/A |
| *Additional systemic therapy* | 0 | N/A | 0 | N/A | N/A | N/A | N/A | N/A |
| **Grade 5** | 0 | N/A |  |  |  |  |  |  |
| *First systemic therapy* | 0 | N/A | 0 | N/A | N/A | N/A | N/A | N/A |
| *Additional systemic therapy* | 1 | Death (reason unknown) | 0 | N/A | N/A | N/A | N/A | N/A |

Supplementary table 3 – *legend*

*IMRT = intensity modulated radiotherapy; N/A = not applicable; R0 = microscopically negative resection margin; R1 = microscopically involved margin; R2 = macroscopic involved margins*
